# COVID-19 and eating disorder and mental health concerns in patients with eating disorders

**DOI:** 10.1186/s40337-021-00437-1

**Published:** 2021-07-02

**Authors:** Julia A. Vitagliano, Grace Jhe, Carly E. Milliren, Jessica A. Lin, Rebecca Spigel, Melissa Freizinger, Elizabeth R. Woods, Sara F. Forman, Tracy K. Richmond

**Affiliations:** 1 Division of Adolescent and Young Adult Medicine, Boston Children’s Hospital, 300 Longwood Ave, Boston, 02115 MA USA; 2 Department of Psychiatry, Harvard Medical School, 25 Shattuck Street, Boston, 02115 MA USA; 3 Institutional Centers for Clinical and Translational Research, Boston Children’s Hospital, 300 Longwood Ave, Boston, 02115 MA USA; 4 Department of Pediatrics, Harvard Medical School, 25 Shattuck Street, Boston, 02115 MA USA

**Keywords:** COVID-19, Eating disorders, Mental health, Comorbidity, Mental health recovery, Adolescents

## Abstract

**Background:**

The Coronavirus (COVID-19) pandemic dramatically transformed daily life for adolescents and young adults, altering social and physical environments. Previous research has shown such shifts in daily life to be especially challenging for people living with eating disorders (ED). However, the extent of this environmental change on ED symptoms and mental health (MH) has been relatively unexplored in patients with EDs. This study examines how young people with EDs feel the COVID-19 pandemic has affected their living environments as well as their ED and MH symptoms and motivation for ED recovery.

**Methods:**

Participants were enrollees in the Registry of Eating Disorders and their Co-morbidities OVER time in Youth (RECOVERY) who responded to an additional survey (*n* = 89) in July 2020 to assess their perceptions of the impact of the COVID-19 pandemic. Participants reported on concerns of their ED worsening due to increased time living in a “triggering environment” due to the pandemic as well as perceived COVID-related changes in intrusive ED thoughts, depression, anxiety, isolation, and motivation to recover. Logistic regression models, adjusted for age and ED diagnosis, examined the association of triggering environment with ED and MH symptoms.

**Results:**

The majority of respondents reported concern for worsening of their ED due to a “triggering environment” (63%). Most reported an increase in ED thoughts (74%), feelings of anxiety (77%), depression (73%), and isolation (80%) they perceived to be related to the pandemic. Nearly one-third reported decrease in motivation to recover (29%) they perceived to be related to the pandemic. After adjusting for age and ED diagnosis, participants who reported concern for worsening of their ED due to a triggering environment had nearly 18 times the odds of decreased motivation to recover (OR 18.1; 95% CI 3.37–97.4, *p* = 0.003) and nearly 24 times the odds of increased ED thoughts (OR 23.8; 95% CI 4.31–131.6, *p* < 0.001) compared to those who did not report concern for worsening of their ED due to a triggering environment.

**Conclusions:**

Our findings demonstrate the perceived negative impact the COVID-19 pandemic has had on the self-reported ED and MH symptoms in patients with EDs, particularly in those who report concern for a negative environmental change. These results underscore the need for heightened monitoring of patients with EDs during the pandemic.

## Plain English Summary

The COVID-19 pandemic and subsequent stay-at-home orders implemented across the world dramatically altered daily life for people of all ages. Previous research has detailed the profound impact the pandemic has had on mental health (MH), particularly among individuals with eating disorders (ED) [22]. This study explored how individuals with eating disorders perceive the COVID-19 pandemic to have impacted their ED symptoms and overall MH. Participants included adolescent/young adults (AYAs) with a diagnosed ED who were given a four-part survey on how they perceive the COVID-19 pandemic to have affected their ED symptoms and treatment and MH (89 participants). AYAs in the study reported feeling that their ED symptoms and MH concerns worsened due to the pandemic. Those who reported concern that their ED would worsen due to a “triggering” living environment were even more likely to report feeling that their ED and MH worsened due to the pandemic. A large percentage of participants also reported a decreased motivation to recover from their ED that they attributed to the pandemic. Overall, individuals with EDs may be at risk for worsening symptoms during the COVID-19 pandemic. Our results underscore the need for increased monitoring of patients with EDs during the pandemic.

## Background

January 2020 marked the first confirmed case of the novel coronavirus (COVID-19) in the United States [1]. Consequently, daily life was dramatically transformed for people of all ages. Following rising cases throughout the U.S., many states implemented stay-at-home advisories and social distancing efforts, strongly recommending physical isolation and at-home quarantine for the health and safety of residents. School-aged children and young adults in 190 countries across the world experienced abrupt changes in their educational and social environments after school closures [2–4]. Not surprisingly, early studies have demonstrated psychological consequences of the COVID-19 pandemic on children and young adults who have been quarantined and/or following stay-at-home recommendations. These consequences include post-traumatic stress symptoms, depression, anxiety, feelings of fear, isolation, and nervousness [5–7].

Patients with eating disorders (ED) may be at risk for the psychosocial implications of COVID-19 restrictions. The stay-at-home advisories and other safety recommendations [8–10] surrounding the COVID-19 pandemic jeopardize in-person ED treatment, potentially risking increased ED symptomatology, social isolation, and stress/anxiety [8–10]. In general, stress has been shown to have serious consequences for patients with EDs, with stress possibly playing a role in ED onset [11–13] and relapse [14]. Previous research suggests undergoing a negative stressful life event significantly predicted relapse for patients with Eds [14]. Given the inherent stressful nature of the COVID-19 pandemic, patients with EDs may be at risk for a negative illness trajectory during the pandemic.

Motivation to recover from an ED is also critical for future ED-related treatment success. Higher levels of motivation for recovery plays a vital role in an individual’s ED illness trajectory, such as positively influencing weight maintenance, treatment response, long-term maintenance of treatment gains, and improvement of ED pathology [15–19]. Thus, identifying how to maintain motivation for recovery, despite the strain of the pandemic, is critical.

The COVID-19 pandemic and its related stressors have greatly influenced all aspects of young peoples’ lives, however, the extent of this influence on patients with EDs is relatively unexplored. Closures of schools and other social distancing dramatically altered youths’ environments and social dynamics, with many teens and young adults forced to abruptly leave school or living environments and return to their childhood homes. While this change in environment may be supportive for some, for others it is potentially distressing [20, 21]. Therefore, it is imperative to understand the role of these environmental disruptions on ED and mental health (MH)-related outcomes.

To address these gaps in research, we examined participant-perceived implications of the COVID-19 pandemic on reported change in ED thoughts, feelings of anxiety, depression and isolation, as well as reported motivation to recover in a sample of adolescents and young adults (AYAs) with EDs. More specifically, we assessed whether environmental changes that were perceived as potentially “triggering”, were associated with self-reported changes in ED/MH symptoms and/or motivation to recover. Overall, we hypothesized the ‘triggering’ environment participants attributed to the COVID-19 pandemic would be positively associated with reported increase in ED intrusive thoughts, feelings of depression, anxiety, isolation, and low motivation to recover from an ED.

## Methods

### Study sample

Our sample is the subset of participants (*n* = 89) in the Registry of Eating Disorders and their Co-morbidities OVER time in Youth (RECOVERY) who completed an optional survey to assess how they perceived the COVID-19 pandemic has affected their daily life. The RECOVERY Study is a longitudinal web-based registry of patients ages 10–27 (average age 17.1 years) seeking ED-related care in an outpatient ED program at Boston Children’s Hospital. RECOVERY seeks to understand the longitudinal experience of youth with EDs, including their trajectories of illness relative to care. A convenience sample of patients with EDs presenting for subspecialty ED-related care (*n* = 161) were recruited between June 2017–August 2020 and asked to complete web-based surveys at regular intervals (every 3 months in year one of participation, every 6 months thereafter). In July 2020, 3 months after the onset of COVID-19 in the United States, we asked participants to complete an additional survey that was specifically COVID-related and was off-cycle relative to the regular survey schedule for participants. The COVID-19 survey asked participants how they perceived COVID-19 affected their treatment, daily life, ED-related symptoms and behavior, and overall well-being. The study participants were not offered any remuneration for completing the optional survey in contrast to the other RECOVERY surveys; the response rate was 56%. The RECOVERY study was approved by the Boston Children’s Hospital Institutional Review Board.

### Survey measures

Survey measures were based on those used by colleagues for an adult sample [9] and were adapted for an AYA population. Likert scale, nominal, open-ended, and ordinal questions were asked in the four-part survey.

### Outcome measures

#### ED/MH related concerns and motivation to recover from ED

Participants were asked “How has the COVID-19 pandemic affected each of the following:” “Feelings of anxiety,” “Feelings of depression,” “Feelings of isolation,” “Intrusive eating disorder thoughts,” and “Motivation to recover from an eating disorder.” A 5-point Likert scale was used with answers ranging from “increased significantly” to “decreased significantly.” Responses were classified into increased (increased significantly, increased somewhat), no effect, and decreased (decreased somewhat, decreased significantly).

### Primary predictor variable

#### Triggering environment

All participants were asked to rate their level of concern to the statement “I have been concerned about worsening of my eating disorder due to increased time living in a triggering environment.” “Triggering environment” was not defined so responses were based on participants’ own definition. A 4-point Likert scale was used, with answers ranging from “not at all concerned” to “very concerned.” Triggering environment was dichotomized into “*any* concern” over ED worsening due to living in a triggering environment (slightly concerned, somewhat concerned, very concerned) vs. “not concerned” (not at all concerned).

### Covariate variables

#### ED diagnosis

Self-reported by patients from a list of eight options (e.g., anorexia nervosa (AN), atypical AN, avoidant restrictive food intake disorder (ARFID), bulimia nervosa, binge-eating disorder, purging disorder, other eating issue(s)/disorder(s), and I don’t know/unsure), allowing patients to choose all diagnoses they felt applied to them. For the purpose of analyses, ED diagnosis was dichotomized into any variant of AN (i.e., AN, atypical AN) vs. other ED.

#### Length of ED treatment

Calculated from date of patient’s ED clinic intake appointment to date of COVID-19 survey completion.

#### Age

Age at COVID-19 survey completion. A dichotomous variable was constructed comparing participants 18 years or older to those under 18 years.

### Statistical analysis

We examined frequencies (percent) for categorical variables and means (standard deviation) for continuous variables. We compared responders to the COVID-19 survey to non-responders from the RECOVERY cohort by demographic factors (age, race/ethnicity and sex) and ED diagnosis using *t-*tests for continuous variables and *χ*^2^ tests for categorical variables. We examined bivariate associations between concern for triggering environment with self-reported changes in: intrusive ED thoughts; feelings of depression; feelings of anxiety; feelings of isolation; and motivation to recover using *χ*^2^ tests. Multinomial logit regression analyses were used to examine the association between concern for triggering environment with self-reported changes in: motivation to recover, feelings of depression, feelings of anxiety, feelings of isolation, and intrusive ED thoughts, adjusting for age and ED diagnosis. All analyses were conducted using SAS (v9.4; Cary, NC) and *p* < 0.05was considered statistically significant.

## Results

### Study sample demographics

Table 1 presents the demographic characteristics of our sample. The 89 RECOVERY participants who completed the COVID-19 survey (*n* = 89 representing 56% response rate), did not differ from the overall RECOVERY participants (*N* = 162) on age at enrollment, race/ethnicity, or sex. However, RECOVERY participants with a restrictive ED were more likely to respond to the COVID-19 survey compared to those with other diagnoses (61% of those with restrictive ED responded compared to 35% of those with other diagnoses, *p* = 0.004). The large majority (84%) of COVID-19 survey respondents self-reported having a restrictive ED. The average age of patients at survey completion was 18.9 and 63% were over 18 (age range of survey participants was 13–27 years). Our study sample was majority female (89%) and White, non-Hispanic (78%). The majority of participants had been in ED treatment for at least 1 year, with more than half of participants (53%) reporting involvement with treatment for 2 years or more.

**Table 1:** Demographic characteristics and eating disorder diagnosis among COVID-19 survey respondents from the RECOVERY study (*N* = 89)

	n (%)			***p***-value
	Overall (*N* = 89)	Concern of eating disorder worsening due to triggering environment		
		Yes (*n* = 56)	No (*n* = 33)	
**Age at survey completion** (years), mean (SD)	18.9 (2.9)	19.2 (2.8)	18.4 (3.2)	0.20
**Age 18 or above at survey completion**	56 (63%)	40 (71%)	16 (48%)	**0.03**
**Female at birth**	80 (89%)	51 (91%)	29 (88%)	0.72
**Race/Ethnicity**				0.20
White, non-Hispanic	69 (78%)	41 (73%)	28 (85%)	
Other race/ethnicity^a^	20 (22%)	15 (27%)	5 (15%)	
**Restrictive ED Diagnosis**	75 (84%)	52 (93%)	23 (70%)	**0.004**
**Length of ED Treatment**				0.49
< 1 year	8 (9%)	6 (11%)	2 (6%)	
1–2 years	34 (38%)	23 (41%)	11 (33%)	
2 years or more	47 (53%)	27 (48%)	20 (61%)	

*SD* standard deviation, *ED* eating disorder

^a^Other race comprised of *n* = 7 Asian, *n* = 6 Multiracial, *n* = 4 Hispanic, *n* = 2 Other race and *n* = 1 Black

### Participants’ perception of the COVID-19 pandemic’s effects on ED/MH symptoms and motivation to recover

A large majority of participants reported the COVID-19 pandemic impacted their ED/MH symptoms (see Table 2), with 73% of participants reported increase in depression, 77% increase in anxiety, 80% increase in isolation, and 74% increase in intrusive ED thoughts.

**Table 2:** Perceived change in eating disorder/mental health concerns due to COVID-19 and concern of eating disorder worsening due to triggering environment

	n (%)			***p***-value
	Overall (***N*** = 89)	Concern of eating disorder worsening due to triggering environment		
		Yes (*n* = 56)	No (*n* = 33)	
**Change in motivation to recover from ED**				**< 0.001**
Decreased	26 (29%)	24 (43%)	2 (6%)	
No effect	40 (45%)	17 (30%)	23 (70%)	
Increased	23 (26%)	15 (27%)	8 (24%)	
**Change in feelings of depression**				**0.03**
Decreased	4 (5%)	1 (2%)	3 (9%)	
No effect	20 (22%)	9 (17%)	11 (33%)	
Increased	65 (73%)	46 (82%)	19 (58%)	
**Change in feelings of anxiety**				0.23
Decreased	2 (2%)	1 (2%)	1 (3%)	
No effect	19 (21%)	9 (16%)	10 (30%)	
Increased	68 (77%)	46 (82%)	22 (67%)	
**Change in feelings of isolation**				0.71
Decreased	2 (2%)	1 (2%)	1 (3%)	
No effect	16 (18%)	9 (16%)	7 (21%)	
Increased	71 (80%)	46 (82%)	25 (76%)	
**Change in intrusive ED thoughts**				**<0.001**
Decreased	6 (7%)	2 (4%)	4 (12%)	
No effect	17 (19%)	2 (4%)	15 (45%)	
Increased	66 (74%)	52 (93%)	14 (42%)	

*ED* eating disorder

Nearly one-third of participants (29%) reported a decrease in their motivation to recover while 45% reported no effect of the pandemic on their motivation to recover.

### Bivariate associations of concerns over triggering environment and ED/MH-related concerns

Many participants reported worrying about their ED worsening due to living in a triggering environment (63%). Such concerns were found to be associated with ED/MH symptoms and participants’ reported motivation to recover (see Table 2). Motivation to recover from the ED, feelings of depression and intrusive ED thoughts were associated with living in a triggering environment (*p* < 0.001, *p* = 0.03, *p* < 0.001, respectively) while feelings of anxiety and isolation were not (*p* = 0.23, *p* = 0.71, respectively).

### Adjusted associations between reported concern for living in a triggering environment and perceived change in ED/MH concerns

Table 3 presents the unadjusted and adjusted associations of concern for ED worsening due to living in a triggering environment and self-reported changes in ED/MH-related concerns. Individuals who reported concern for ED worsening due to living in a triggering environment had 18 times the odds of reporting a perceived pandemic-related decrease in their motivation to recover (95% CI 3.37–97.4, *p* = 0.003) compared to those who denied concerns of ED worsening due to living in a triggering environment. Those who reported concerns about their ED worsening due to living in a triggering environment had nearly 24 times the odds of reporting a perceived pandemic-related increase in intrusive ED thoughts (95% CI 4.31–131.6, *p* < 0.001) compared to those not concerned. Finally, those who reported concern for their ED worsening due to living in a triggering environment had increased odds of reporting worsening feelings of depression; this association, however, was attenuated after adjusting for age and diagnosis. Associations between triggering environment and feelings of isolation and anxiety were not significant.

**Table 3:** Unadjusted and adjusted odds of perceived change in eating disorder/mental health symptoms due to reported triggering environment (*N* = 89)

Outcome	Unadjusted			Adjusted^**b**^		
	Odds Ratio (95% CI)^**a**^		***p***-value	Odds Ratio (95% CI)^**a**^		***p***-value
	Decreased vs. No Change	Increased vs. No Change		Decreased vs. No Change	Increased vs. No Change	
**Change in motivation to recover from ED**	**16.2 (3.37, 78.3)**	2.54 (0.88, 7.34)	**0.002**	**18.1 (3.37, 97.4)**	2.39 (0.78, 7.35)	**0.003**
**Change in feelings of depression**	0.41 (0.04, 4.62)	**2.96 (1.06, 8.29)**	**0.043**	0.35 (0.03, 4.28)	1.89 (0.61, 5.90)	0.25
**Change in feelings of anxiety**	1.11 (0.06, 20.5)	2.32 (0.83, 6.53)	0.26	0.62 (0.03, 12.6)	1.31 (0.39, 4.34)	0.81
**Change in feelings of isolation**	0.78 (0.04, 14.8)	1.43 (0.48, 4.31)	0.76	0.32 (0.01, 7.04)	0.68 (0.18, 2.59)	0.73
**Change in intrusive ED thoughts**	3.75 (0.40, 35.5)	**27.8 (5.69, 136.4)**	**<0.001**	3.74 (0.38, 37.2)	**23.8 (4.31, 131.6)**	**<0.001**

*ED* eating disorder

^a^Odds ratios reported are for the primary predictor “concern of ED worsening due to living in a triggering environment” (yes vs. no) predicting each outcome

^b^Adjusted for age and restrictive diagnosis

## Discussion

Our findings demonstrate the urgent perceived implication of the COVID-19 pandemic by youth with EDs. The large majority of participants reported the COVID-19 pandemic affected their worsening ED/MH symptoms, with many also reporting declining motivation to recover they attributed to the pandemic. Individuals also reported high degree of environmental disruption, with well over half of participants expressing concern that their ED would worsen due to living in a triggering environment. The report of living in a triggering environment was further associated with marked increased risk of reporting worsening ED/MH symptoms relative to no report of concern for a triggering environment. More specifically, concerns for their ED worsening due to increased time living in a triggering environment were associated with increased intrusive ED thoughts and decreased motivation to recover.

As hypothesized, the majority (over 70%) of AYA patients with EDs reported feeling the COVID-19 pandemic affected them by increasing intrusive ED thoughts, anxiety, depressive symptoms, and social isolation. This is consistent with other studies of the impact of COVID-19 on patients with EDs. Rates of ED symptoms (e.g., dieting, excessive exercise, purging), anxiety, and depression among AYA populations with EDs have been consistently higher during the pandemic compared to other years, as evidenced by a significant increase in the number of helpline calls and online instant chats with their National ED Centre in Canada [22]. Termorshuizen et al. (2020) found that AYA participants with AN reported increased restrictive eating and fear about finding foods to follow their meal plans while those with bulimia nervosa and binge eating disorder reported increased binge-eating behaviors [9]. Another study found that more adolescents and adults with AN agreed rather than disagreed that their ED symptoms and sadness had worsened [10]. Research suggests that individuals with EDs may be experiencing worsening ED symptoms due to feelings of social isolation, which leads to loneliness, fewer distractions, more time to think about food, and increased opportunity to engage in disordered behaviors [21, 23].

Regarding individuals’ motivation for recovery in the context of COVID-19, rates of reporting a decrease versus an increase in their motivation were similar (29 and 26%, respectively). Recent studies have identified potential factors that contribute to an increase in motivation for recovery, including increase in social support that challenges ED behaviors [9], positive influence from others (e.g., recovered role models, advocacy groups, mentors), support for pro-recovery beliefs and lifestyle changes, and non-judgmental comments around weight or eating [20]. There is limited understanding of what contributes to decreased motivation during the COVID-19 pandemic. It is possible that the worsening quality of the parent-adolescent relationship or avoidance of negative or distressing emotions may be associated with decreased motivation to recover among adolescents with EDs [18, 24].

The present study found that more than a half of the participants reported feeling concerned that their ED would worsen due to increased time living in a triggering environment. This finding is consistent with Termorshuizen et al.’s (2020) study on COVID-19-related concerns and EDs, which showed that about 58% of their participants reported concerns about worsening of ED symptoms due to increased time living in a triggering environment [9]. While “triggering environment” was not defined in the survey measure we adapted from colleagues [9], some examples included social media content on weight gain during quarantine, a lack of structure, and being at home all day [9]. Given the age range of our population, with many college-aged participants, the worry about a triggering environment could also be related to an abrupt return to one’s childhood home after being away at a university/college or living independently. Other COVID-19-related stressors that may further exacerbate ED symptoms include changes in daily schedule or routines [21, 25], increase in financial stress [5], food insecurity [26], as well as family conflicts [10, 27]. Living and spending a large amount of time at home may be triggering, as some adults who had recovered from an ED had felt judged by family at home [20]. Family dynamics are important to consider given the age of the present study’s participants, as many returned home or were required to spend more time at home during the lockdown.

Our study highlights the impact of environmental stressors on individuals’ ED symptoms and motivation for recovery. We found that individuals who were concerned that their ED would worsen due to increased time living in a triggering environment were 18 times more likely to report a decrease in their motivation to recover and nearly 24 times more likely to experience an increase in intrusive ED thoughts compared to those who denied any concerns about ED due to their environment. This is consistent with recent findings that lockdown restrictions, disrupted routines, increased time spent in a triggering environment, and decreased ability to engage in activities (including exercise) may lead to individuals’ frustration and feelings of restlessness [9, 25]. These individuals may also experience beliefs that they do not need to eat as much as they did pre-COVID, which may further be reinforced by media attention to weight gain during lockdown for the general public [25, 28]. Individuals with EDs may also experience loneliness, social isolation, decreased social support, and a lack of distractions related to COVID-19, which can lead to greater focus or rumination on food and disordered eating behaviors [21, 23] and greater difficulty with coping with ED cognitions [29].

These findings suggest that stressful environments—such as one brought upon by the current COVID-19 pandemic—may affect motivation to recover and ED/MH symptoms. The literature on COVID-19 has demonstrated the negative impact of COVID-19 on MH concerns (anxiety, depression, etc.). Our study examines the environmental changes related to the pandemic and their unique effects on ED/MH symptoms and motivation for recovery. To our knowledge, the present study is one of the first to look at COVID-19’s impact on ED/MH symptoms and motivation to recover among an AYA population with an ED.

Despite its aforementioned strengths, the present study is not without limitations. Though it was made clear to patients that this additional survey was to assess the effects of COVID-19 on their ED, we cannot make a causal conclusion that the many changes related to COVID-19 pandemic caused these ED and MH changes. Additionally, “triggering environment” was not defined in the survey measure used. Participants were asked if they had concerns about their ED worsening due to increased time living in a triggering environment and thus, respondents may have retrospectively chosen this as a reason to explain why their ED has worsened. Although the majority of patients from our full sample responded to the survey (56%), it is still unclear how the remaining participants would have influenced our results. Subsequently, our moderate response rate was likely partially due to these being additional surveys sent off-cycle with no remuneration in order for data to be collected in a timely manner. Participants who may have found the pandemic particularly challenging could have been more likely to respond to this off-cycle survey, potentially biasing our results. Responders only differed from the non-responders on ED diagnosis, although it should be noted that ED diagnoses were self-reported. Additionally, the majority of our patients were white and/or had a restrictive ED diagnosis and thus, the generalizability of our findings for patients with other ED diagnoses (e.g. binge-eating disorder, bulimia nervosa) and other self-identified races and ethnicities may be limited. Overall, our findings cannot be generalized to other samples. Due to small sample size, we carefully considered which variables to adjust for to avoid overfitting the model. Despite wide confidence intervals due to sample size, odds ratios were very large, indicating a compelling effect of the extent the COVID-19 pandemic has been perceived to have impacted patients living with EDs. Additionally, a sensitivity analysis collapsing “triggering environment” into “slightly concerned”/“no concern” vs. “somewhat concerned”/“very concerned” yielded similar overall results in adjusted analyses to the findings of the primary analysis reported in the results and tables. In order to provide a broader picture of how the COVID-19 pandemic impacted patients with EDs, future research should focus on the perspective of caregivers, as caregiver burden and stress may indirectly affect the ED and MH concerns of patients with EDs. Our findings illustrate worsening ED/MH concerns perceived to be due to the COVID-19 pandemic in a subsample of patients with EDs and offers a possible explanation in environments impacted by the pandemic.

## Conclusions

As the present study showed, the COVID-19 pandemic and its related environmental stressors are perceived by our sample of youth with EDs to have negatively impacted ED-and MH-related concerns. Understanding the COVID-19-related, deleterious environmental effects on ED symptoms and motivation for recovery is crucial in order for providers to support patients in coping with the sudden disruptions to their daily living with uncertain timeframe.

## Data Availability

The datasets generated and/or analyzed during the current study are not publicly available due to patient confidentiality and the commitment given to all participants in protecting their identity. Data are available de-identified from the corresponding author on reasonable request and IRB approval.

## Abbreviations

ED: Eating disorder
MH: Mental health
AYA: Adolescent/Young adult
AN: Anorexia nervosa
ARFID: Avoidant restrictive food intake disorder
